# Classifications of good versus poor outcome following knee arthroplasty should not be defined using arbitrary criteria

**DOI:** 10.1186/s12891-020-03583-w

**Published:** 2020-09-10

**Authors:** Daniel L. Riddle, Levent Dumenci

**Affiliations:** 1 Departments of Physical Therapy, Orthopaedic Surgery and Rheumatology, Virginia Commonwealth University, Richmond, VA USA; 2 Department of Epidemiology and Biostatistics, 1301 Cecil B. Moore, Ave., Ritter Annex, Room 939, Temple University, Philadelphia, PA 19122 USA

**Keywords:** Knee, arthroplasty, outcome, pain, function

## Abstract

A recently published paper by te Molder and colleagues in BMC Musculoskeletal Disorders confirmed prior reports indicating that definitions of good versus poor outcome cutoff scores for relevant knee arthroplasty outcomes including pain and function are heterogeneous and that this heterogeneity prevents generalizable inferences. In this Correspondence, we highlight an additional and, in our view, a more important problem with the substantial literature on this topic. There also is high homogeneity in that all studies relied on arbitrarily defined cutoff scores to differentiate good versus poor outcome. We discuss this problem and propose a method to avoid repeating the same problem in future studies designed to group patients into those with good versus those with poor outcome following knee arthroplasty.

## Main text

The systematic review by te Molder and colleagues [1] summarized various methods used by investigators to dichotomize outcomes of patients with knee arthroplasty (KA) as either good or poor. There are important reasons for wanting to know if a patient’s KA outcome is good or poor. For example, interventions to improve outcome can be specifically designed and targeted to patients fitting the poor outcome phenotype. The dilemma with categorizing outcome, as te Molder et al. and others [2, 3] have noted, is that definitions of good versus poor outcome vary substantially across the many studies that have attempted to categorize outcomes following KA. Variation precludes consensus and prevents meaningful comparisons across study cohorts. We noted an additional problem with evidence classifying outcome as good or poor [4]. Definitions of good versus poor outcome are grounded in the use of arbitrary cutoff values, whether based on final outcome score, percent or absolute change from baseline or the Minimal Clinically Important Difference (MCID) family of change indicators.

The main conclusion of the study by te Molder and colleagues was that there was substantial heterogeneity in the 47 definitions of good versus poor KA outcomes. In our view, te Molder et al. should also have focused on implications related to the homogeneity of these 47 definitions. All studies in the review used the cutoff method to determine good versus poor outcome. Cutoff scores are, by definition, arbitrary. Supplemental file 3 in the study by te Molder et al. [1] provides a partial list of definitions used to establish arbitrary cutoff scores (including two of our prior studies [5, 6]). For example, Brander and colleagues indicated that a 0 (no pain) to 100 (worst pain imaginable) visual analogue pain scale of > 40 indicated a poor pain outcome [7]. This cutoff is arbitrary.

Over three decades ago, researchers and clinicians were warned about the arbitrary nature of the cutoff method for clinical decision making and proposed latent class analysis as a scientifically defensible alternative [8]. Recent methodological developments also have been extensively documented [9]. In 2011, we further elaborated on why the cutoff method should not be used to determine patient groupings in scientific research, developed methods originating from discrete latent variable modeling approaches to circumvent problems associated with the arbitrary cutoff method, and provided multiple examples using real-life data to illustrate how new methods could be used to answer scientific questions [10]. In 2019, we used methods originating from a longitudinal discrete latent variable modeling framework to define poor versus good outcomes in KA [4]. For reasons that were unclear to us, given that it met inclusion criteria by te Molder and colleagues, our 2019 study [11] was not included in the review. This latent variable modeling method does not rely on biased good versus poor cutoffs but rather on statistical modeling that is free of arbitrary decision-making.

The cutoff method is an impediment to scientific progress. If we continue to overlook homogeneity, and don’t acknowledge that this evidence relies on arbitrary cutoff scores, we will keep using arbitrary cutoff scores to define poor outcome in KA. Going down this road would lead to even more studies that rely on arbitrary cutoffs and we’ll have made no progress. In our view, the answer to the lack-of-consensus problem posed by te Molder et al. for defining good versus poor outcome in KA is not to continue relying on arbitrary cutoff scores. Instead, we should rely on a non-biased statistical model-based approach to categorizing good versus poor outcome [11].

Once the cutoff method is replaced with model-based approaches, we suggest the following strategy: Researchers focus on factors that matter most as the sources of outcome variability. For example, what constitutes the KA outcome (e.g., self-reported knee pain, function, health-related quality of life)? Whose perspective(s) should be captured (e.g., patients, relatives, surgeons, or a combination)? What are the optimal time point(s) for measuring outcome (e.g., 2 weeks before and after KA, and four additional times over subsequent 2 years)? What are the key predictors of good versus poor outcome classes? We contend that a coordinated consensus-based strategy like the one described above is needed to shift the paradigm of this type of work and advance the science of good versus poor outcome identification in KA.

**Abbreviations**

KA: Knee arthroplasty; MCID: Minimal Clinically Important Difference

**Acknowledgements**

N/a

**Authors’ contributions**

DLR and LD each contributed to the original draft, the revisions and both approved the final version.

**Funding**

No funding was obtained for the paper.

**Availability of data and materials**

N/a

**Ethics approval and consent to participate**

N/a

**Consent for publication**

N/a

**Competing interests**

The authors declare that they have no competing interests.
