# Supplementary material for: Classifications of good versus poor outcome following knee arthroplasty should not be defined using arbitrary criteria
Source: BMC Musculoskelet Disord. 2020 Sep 10;21:604. doi: 10.1186/s12891-020-03583-w (PMC7488551; doi:10.1186/s12891-020-03583-w)
Supplement: Related texts

# Reply: Author’s response

**Authors:** Malou E. M. te Molder, José M. H. Smolders, Petra J. C. Heesterbeek, Cornelia H. M. van den Ende

**Affiliations:** 3 grid.452818.2 0000 0004 0444 9307 Department of Orthopedic Research, Sint Maartenskliniek, Nijmegen, The Netherlands; 4 grid.10417.33 0000 0004 0444 9382 Department of Orthopaedic Surgery, Radboud University Medical Center, Nijmegen, The Netherlands; 5 grid.452818.2 0000 0004 0444 9307 Department of Orthopedic Surgery, Sint Maartenskliniek, Nijmegen, The Netherlands; 6 grid.452818.2 0000 0004 0444 9307 Department of Rheumatology Research, Sint Maartenskliniek, Nijmegen, The Netherlands; 7 grid.10417.33 0000 0004 0444 9382 Department of Rheumatology, Radboud University Medical Center, Nijmegen, The Netherlands

Thank you for giving us the opportunity to write a response to the correspondence “Classification of good versus poor outcome following knee arthroplasty should not be defined using arbitrary criteria”.

We thank Riddle *et al* for their interest and critical assessment of our inventory review in which we summarized definitions of poor response to total knee arthroplasty (TKA). Riddle *et al* suggest that we should have focused on implications related to the arbitrary and homogeneous use of cutoff points. Instead, Riddle *et al* strongly recommend to rely on a model-based approach to define poor response to TKA. Several model-based approaches are available to identify subgroups with different growth curves. We acknowledge the value of those models. However, a major limitation of these types of models is that membership of poor and good outcome classes can only be determined afterwards and that results with regard to membership of classes cannot be transferred to other study populations.

We fully agree that a drawback of dichotomizing data is data reduction and that a continuous measure is more sensitive to change, and, therefore, more useful on individual level and in clinical decision making. Mixture models can provide more in-depth insight in the course of outcome over time and its determinants. However, to allow comparisons of the prevalence of poor responders to TKA across hospitals, countries, and over time, a strict definition is necessary with clearly defined criteria and thresholds. For this purpose, a dichotomous outcome is more appropriate while the use of mixture models is preferred if the purpose is to gain insight in factors underlying outcomes over time.

The second remark relates to the reason why the 2019 study by Dumenci *et al* [4] was not included in the review. As the authors indicated in their correspondence, inclusion criteria for our inventory review focused on predefined dichotomized cutoffs to define poor outcome and, therefore, studies including model-based approaches (such as the study by Dumenci *et al*) were not included.

Nevertheless, we thank Riddle *et al* for their suggested strategy to focus on factors, perspective(s) and optimal time point(s) for measuring good versus poor outcome in TKA. The intended strategy of our project is exactly what Riddle *et al* proposed. We use the results of our inventory review and an ongoing qualitative study in patients as well as health care providers to focus on relevant concepts underlying a poor response to TKA. Once the relevant concepts have been identified, we can start the discussion among panelists of a subsequent Delphi study. The ultimate aim of our project is to reach consensus on a definition of poor response to TKA after which we, hopefully, can properly compare the prevalence of poor responders across hospitals and countries.
